# *Bacillus velezensis* Enhances Rice Resistance to Brown Spot by Integrating Antifungal and Growth Promotion Functions

**DOI:** 10.3390/ijms27031455

**Published:** 2026-02-01

**Authors:** Elizabeth B. E. Pires, Maira S. Tique Obando, Luis Janssen, Bergmann M. Ribeiro, Odaiza F. Souza, Marcelo L. Dias, Luís O. Viteri Jumbo, Rodrigo R. Fidelis, Gil R. Santos, Raimundo N. C. Rocha, Guy Smagghe, Tito Bacca, Eugenio E. Oliveira, Rudolf Haumann, Raimundo W. S. Aguiar

**Affiliations:** 1 Programa de Pós-Graduação em Biotecnologia, Universidade Federal do Tocantins (UFT), Gurupi 77402-970, TO, Brazil; elizabethpires11.22@gmail.com (E.B.E.P.);; 2 Departamento de Biologia Celular, Universitdade de Brasília, Brasília 70297-400, DF, Brazil; 3 Programa de Pós-graduação em Biodiversidade e Biotecnologia—Rede Bionorte, Universidade Federal do Tocantins (UFT), Gurupi 77402-970, TO, Brazil; 4 Programa de Pós-graduação em Produção Vegetal, Universidade Federal do Tocantins, Gurupi 77410-530, TO, Brazil; 5 Embrapa Arroz e Feijão, Santo Antônio de Goiás 75375-000, GO, Brazil; 6 Institute of Entomology, Guizhou University, Guiyang 550025, China; 7 Department of Biology, Vrije Universiteit Brussel (VUB), 1050 Brussels, Belgium; 8 Facultad de Ingeniería Agronómica, Universidad del Tolima, Ibagué 730001, Tolima, Colombia; 9 Departamento de Entomologia, Universidade Federal de Viçosa, Viçosa 36570-900, MG, Brazil; 10 Department of Bioprocess Engineering, Institute of Food Science and Biotechnology, University of Hohenheim, 70599 Stuttgart, Germany

**Keywords:** *Bipolaris oryzae*, biological control, biosynthetic gene clusters, lipopeptides, antifungal activity

## Abstract

Brown spot, caused by the seedborne fungus *Bipolaris oryzae*, remains a major constraint in rice production. Here, we used in vitro and in vivo assays to evaluate the biocontrol potential of three *Bacillus* strains (*Ba. cereus* OQ725688.1, *Ba. velezensis* OP938696.1, and *Ba. subtilis* OP937353.1) against *Bi. oryzae* in two rice cultivars (“Rubelita” and “Predileta”). *Ba. cereus* showed the highest in vitro mycelial inhibition (≈95%), whereas *Ba. velezensis* was the most effective under greenhouse conditions, reducing disease severity by up to 60% and increasing seedling vigor by 51% compared with infected controls. “Predileta” showed the strongest response to bacterial treatment, maintaining severity scores below 2 even under high inoculum pressure. Functional assays confirmed that all strains displayed amylolytic, catalase, and phosphate-solubilizing activities, with *Ba. velezensis* uniquely expressing strong cellulase and protease activities. Genome analysis of *Ba. velezensis* OP938696.1 revealed multiple biosynthetic gene clusters for antifungal polyketides and lipopeptides. These integrated biochemical and genomic traits demonstrate the novelty and potential of this Neotropical strain as a multifunctional agent capable of suppressing *Bi. oryzae* while enhancing rice seedling performance. Incorporating such a native strain into seed and soil management offers a sustainable strategy for rice protection in Neotropical systems.

## 1. Introduction

Rice, *Oryza sativa* L., represents one of the principal pillars of global food security, serving as the staple food for more than half of the world’s population [1]. However, its productivity is increasingly threatened by fungal diseases, among which brown spot, caused by *Bipolaris oryzae*, is of particular concern. This disease compromises grain integrity and, in severe outbreaks, can result in yield losses approaching 90% [2,3]. The catastrophic impact of such epidemics was historically demonstrated during the Great Bengal Famine [4], emphasizing the persistent vulnerability of rice production to phytopathological stresses. Recent regional outbreaks in Asia, driven by climatic conditions that favor *Bi. oryzae* proliferation, reaffirm the continued relevance of this pathogen in the context of global climate change and growing food demand [5,6,7].

Efforts to mitigate brown spot disease have largely focused on developing resistant rice cultivars [8,9]. However, the durability of host resistance remains inconsistent, frequently compromised by the pathogen’s high genetic variability and rapid adaptive capacity. The dynamic interplay between *O. sativa* defense responses and *Bi. oryzae* virulence determinants remains poorly understood at the molecular level, impeding the stable deployment of resistance genes across environments [10,11,12]. Advances in genomics and transcriptomics have revealed the complex regulatory networks underlying plant–pathogen interactions, yet the functional mechanisms through which *Bi. oryzae* suppresses host immunity or how rice modulates its defense signaling pathways, particularly involving salicylic acid (SA), jasmonate (JA), and ethylene, are still only partially elucidated [3,13]. Understanding these molecular dynamics is crucial for designing durable resistance strategies and integrating them with sustainable crop management systems.

Although synthetic fungicides remain the most widely used control method, their intensive and often indiscriminate application raises severe ecological and toxicological concerns. Overreliance on fungicides has accelerated the emergence of resistant fungal strains, thereby reducing product efficacy and complicating long-term disease control [14,15]. Moreover, fungicide residues in soils and food products contribute to environmental contamination and pose potential human health risks [16,17,18]. For example, recent assessments in Brazil, which is one of the world’s largest pesticide consumers, have documented concerning levels of chemical residues in commonly consumed foods [19,20]. Consequently, there is an urgent need to identify and develop environmentally sustainable and biologically based alternatives that can ensure effective disease control without compromising public health or ecosystem stability.

In this context, the exploration of biological control agents (BCAs) has emerged as a promising avenue within integrated disease management frameworks. These biotechnological approaches seek to reduce dependence on synthetic inputs while enhancing crop resilience through the natural antagonism between beneficial and pathogenic microorganisms [19,21]. Increasingly, beneficial microorganisms are being recognized for their capacity to modulate plant defense responses at the molecular level and for their contribution to the development of agricultural bioinputs [22,23,24,25,26,27]. Their interactions with host plants often involve complex molecular signaling, including the modulation of phytohormone pathways, the activation of defense-related gene expression, and the production of bioactive secondary metabolites that inhibit pathogen growth.

Among the most studied BCAs, *Bacillus* spp. have received particular attention due to their remarkable metabolic versatility and ecological adaptability. Members of this genus produce a wide array of antimicrobial compounds, such as lipopeptides (iturins, fengycins, surfactins), polyketides, and bacteriocins, that can disrupt fungal membrane integrity or interfere with pathogen signaling [24,26,28,29,30,31,32]. Their ability to form endospores confers exceptional persistence under environmental stress, while their efficient rhizosphere colonization facilitates sustained interactions with plant roots [26]. Moreover, several *Bacillus* strains are known to trigger induced systemic resistance (ISR) through the activation of defense-related pathways, including those mediated by JA and ethylene [33,34,35]. Beyond pathogen suppression, *Bacillus* spp. frequently acts as plant growth-promoting (PGP) rhizobacteria, enhancing nutrient acquisition, stimulating phytohormone production, and improving tolerance to abiotic stresses [26,29,36,37,38,39,40]. However, little is known about how rice genotypes differ in their responsiveness to Bacillus-mediated biocontrol and defense activation.

In the present study, we aimed to functionally characterize *Bacillus* spp. strains with potential for biological control of *Bi. oryzae* in rice. We evaluated their antagonistic activity in two rice varieties, assessing both disease severity and shoot biomass accumulation. Furthermore, we performed phylogenetic analyses based on 16S rRNA gene sequences to explore potential relationships between genetic relatedness and biocontrol efficiency. By integrating microbiological, phytopathological, and molecular approaches, this study seeks to elucidate the biological basis of *Bacillus*-mediated suppression of *Bi. oryzae* and its influence on plant physiology. Ultimately, our findings aim to contribute to the rational selection of microbial strains for the formulation of biofertilizers and biorational fungicides, advancing the principles of molecularly informed and sustainable agriculture.

## 2. Results

### 2.1. Subsection Morphology of Bi. oryzae Under Rice Infection Conditions

**Figure 1:**
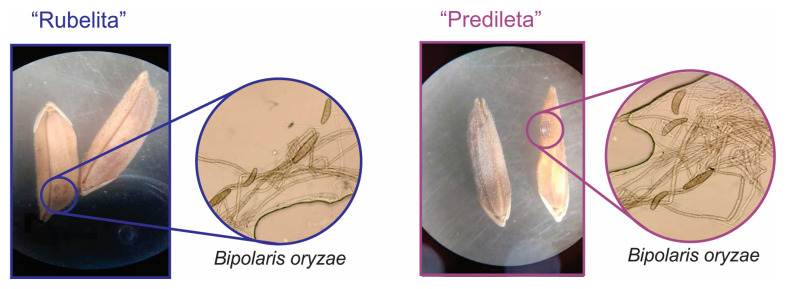
Detection of *Bipolaris oryzae* in the rice, *Oryza sativa*, cultivars “Rubelita” and “Predileta”. The inlet images show micrographs revealing fungal conidia and hyphae on the seed hulls, confirming infection in both cultivars.

Microscopic and macroscopic analyses confirmed successful colonization of rice grains by *B. oryzae* in both cultivars (Figure 1). In the cultivar “Predileta” fungal growth was more pronounced, with dense hyphal networks and abundant conidia adhering to the grain surface. In contrast, the “Rubelita” cultivar (Figure 1) exhibited visibly lower hyphal density and fewer conidia, suggesting reduced colonization.

These observations indicate a differential host response to infection, with “Predileta” showing higher susceptibility and “Rubelita” displaying a more restrictive or tolerant phenotype. Such cultivar-dependent differences highlight the influence of host genotype on pathogen colonization dynamics and underscore the potential of “Rubelita” as a valuable genetic resource for brown spot resistance breeding.

### 2.2. Antagonistic Activity of Bacillus spp. Strains Against Bi. oryzae in In Vitro Assays

**Figure 2:**
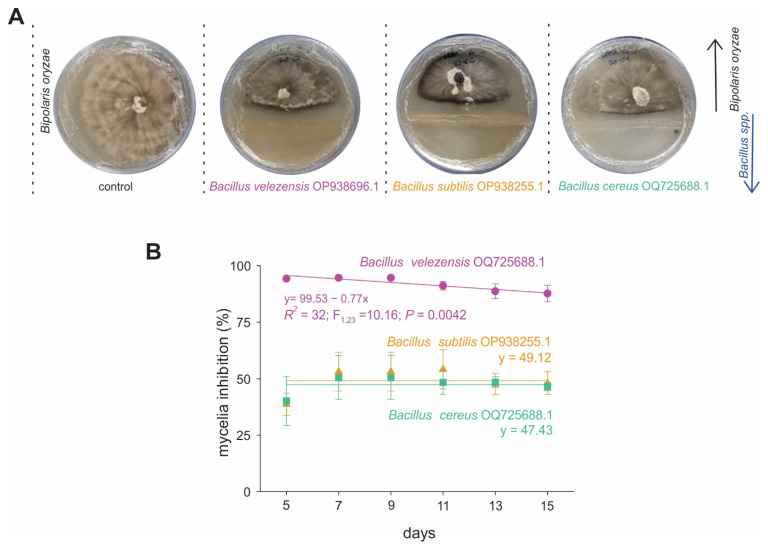
In vitro antagonistic activity of *Bacillus* spp. against *Bi. oryzae* in dual-culture assays. (**A**) Representative illustration of activities of different strains of *Bacillus* spp. (**B**) Inhibition mean (±SE) of *Bi. oryzae* over time post-inoculation. Symbols showed the results of four replicates.

In dual-culture assays, all *Bacillus* spp. strains exhibited clear antagonistic activity against *Bi. oryzae*, significantly reducing mycelial growth compared with the untreated control (Figure 2). Among the tested strains, *Ba. velezensis* (Figure 2B) demonstrated the strongest inhibition, maintaining suppression levels consistently above 90% throughout the experimental period. The other *Bacillus* strains displayed moderate but stable inhibitory effects, with mean growth suppression rates of approximately 49% and 47%, respectively.

These results demonstrate notable variability in antifungal potential both within and among *Bacillus* strains, reinforcing the importance of strain-specific functional characterization. The pronounced antagonistic performance of *Ba. velezensis* suggests a distinct ability to interfere with *Bi. oryzae* growth, potentially linked to the production of bioactive secondary metabolites such as lipopeptides or polyketides, which are well-documented antifungal compounds within the *Ba. subtilis* species complex.

### 2.3. Phylogenetic Characterization of Bacillus Strains

**Figure 3:**
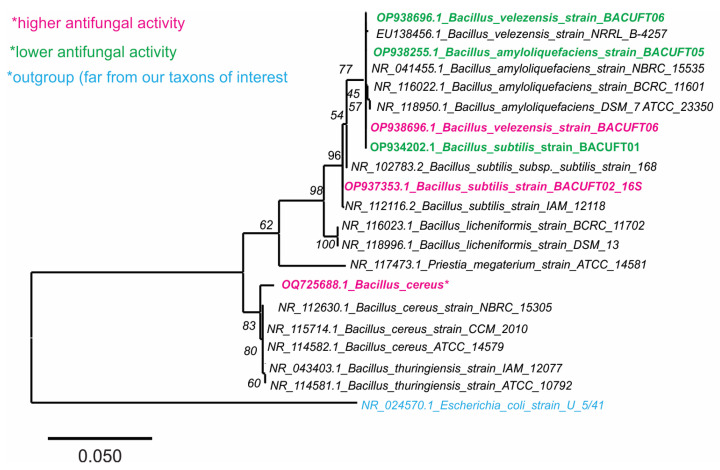
Phylogenetic tree based on 16S rRNA gene sequences of *Bacillus* spp. strains. Strains are highlighted with colors according to their antifungal activity: magenta indicates higher activity, green indicates lower activity, and blue indicates a group far from our taxon of interest. Evolutionary distances are represented by the scale bar (0.050 substitutions per nucleotide position).

Phylogenetic analysis based on 16S rRNA gene sequences positioned all strains within the genus *Bacillus*, showing strong similarity to members of the *Ba. subtilis* complex (Figure 3). Two strains (GenBank accessions OP938696.1 and OP937353.1) clustered together in a well-supported clade containing both *Ba. subtilis* and *Ba. velezensis* reference strains, with high bootstrap values confirming their taxonomic assignment within this group. A third isolate (OQ725688.1) grouped separately within the *Ba. cereus* clade, also supported by robust phylogenetic confidence. These findings corroborate the molecular identity of the strains and reveal taxonomic diversity among the strains tested. The clustering pattern aligns with known phylogenetic relationships in the *Bacillus* genus and supports the identification of *Ba. velezensis* as the strain exhibiting the highest antifungal potential in vitro.

### 2.4. Effects of Bacillus spp. on Rice Development and Brown Spot Severity

**Figure 4:**
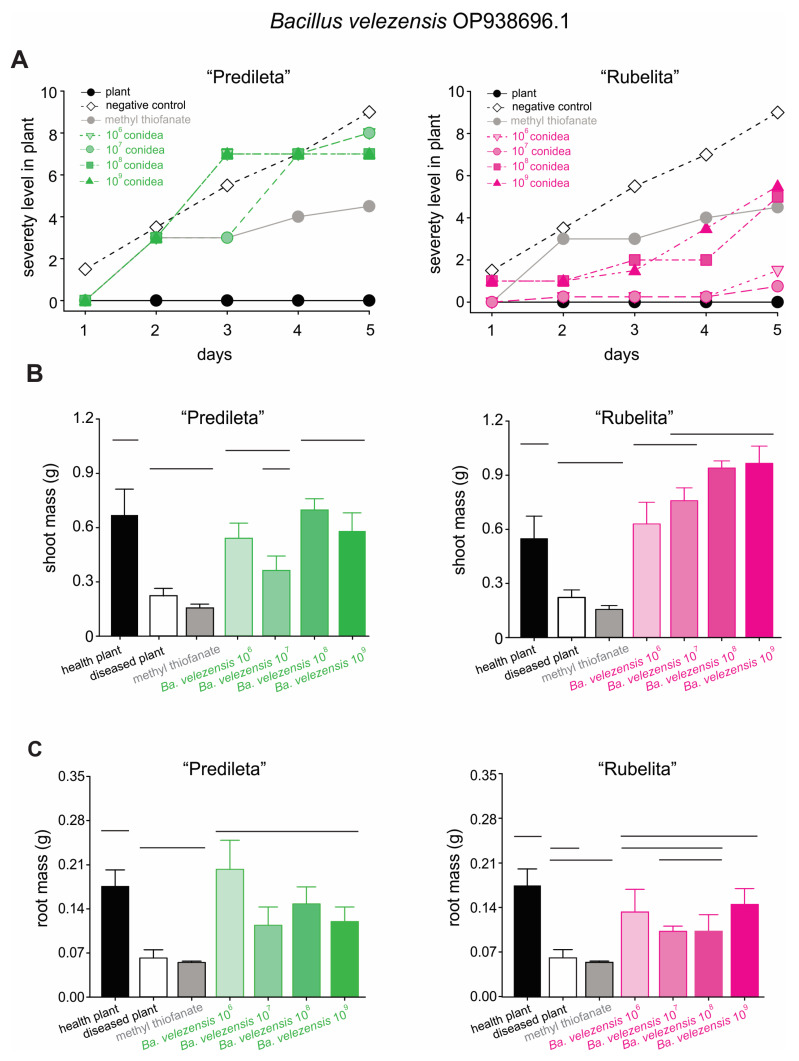
Brown spot disease severity (**A**), shoot biomass (**B**), and root biomass (**C**) of rice plants of the “Predileta” and “Rubelita” cultivars treated with *Bacillus velezensis* OP938696.1 (**C**). (**A**) Symbols show the means of four replicates. (**B**,**C**) Bars represent mean ± SE of four replicates. Horizontal lines above bars indicate no significant differences (Duncan test, *p* < 0.05).

The application of *Bacillus* strains markedly reduced brown spot severity in both rice cultivars when compared with the untreated control (Supplementary Figure S1). Disease suppression was evident across all *Bi. oryzae* inoculum concentrations, although the magnitude of the effect varied depending on the bacterial isolate and rice cultivar. In the untreated control, the highest inoculum level (10^9^ conidia per mL) produced the maximum severity score (5), particularly in the cultivar “Predileta”, which consistently exhibited greater disease intensity across all concentrations (Supplementary Figure S1). In contrast, “Rubelita” maintained markedly lower severity scores, indicating higher tolerance to the pathogen (Supplementary Figure S1). Treatment with *Ba. velezensis* OP938696.1 consistently reduced brown spot severity and promoted plant growth in both rice cultivars (Figure 4A). In “Predileta”, symptom severity increased with rising inoculum pressure, although values remained substantially lower than those observed in untreated controls. In contrast, “Rubelita” exhibited near-complete protection, maintaining severity scores below 2 even at the highest inoculum level (10^9^ conidia mL^−1^).

Regarding plant growth, across all tested strains and concentrations, plants treated with *Bacillus* spp. exhibited higher shoot mass compared to diseased controls and those treated with methyl thiophanate (Supplementary Figure S2). The positive effect was particularly evident at higher bacterial concentrations (10^8^–10^9^ CFU mL^−1^), where shoot biomass approached or matched that of healthy plants (Supplementary Figure S2). Among the evaluated strains, *Ba. velezensis* OP938696.1 exhibited the most pronounced growth-promoting effect, especially in the “Rubelita” cultivar (Figure 4B), suggesting superior performance of this strain under the tested conditions. *Bacillus velezensis* OP938696.1 significantly enhanced shoot biomass under disease pressure, with concentration-dependent effects that were most pronounced at 10^8^–10^9^ CFU mL^−1^. Shoot mass increased by approximately 120–180% in “Predileta” and up to 280% in “Rubelita” relative to diseased plants, in several cases approaching values observed in healthy controls (Figure 4B).

For root biomass results, both “Rubelita” and “Predileta” cultivars exhibited substantial reductions in root mass under disease conditions, whereas all *Bacillus* treatments promoted recovery of belowground growth compared with diseased and methyl thiophanate-treated plants (Supplementary Figure S3). In “Predileta”, *Ba. cereus* OQ725688.1 induced the strongest stimulation of root biomass, increasing root mass by approximately 140–190% relative to diseased plants, particularly at 10^6^ CFU mL^−1^ (Supplementary Figure S3). In contrast, in “Rubelita”, *Ba. velezensis* OP938696.1 produced the most consistent and pronounced enhancement, with increases ranging from 40% to 110% depending on bacterial concentration (Figure 4C). Although the magnitude of response was lower than that observed for shoot biomass, both *Ba. cereus* and *Ba. velezensis* demonstrated clear plant growth-promoting activity, with strain-specific and cultivar-dependent effects.

### 2.5. Biochemical and Functional Characterization of Bacillus spp. Strains

**Figure 5:**
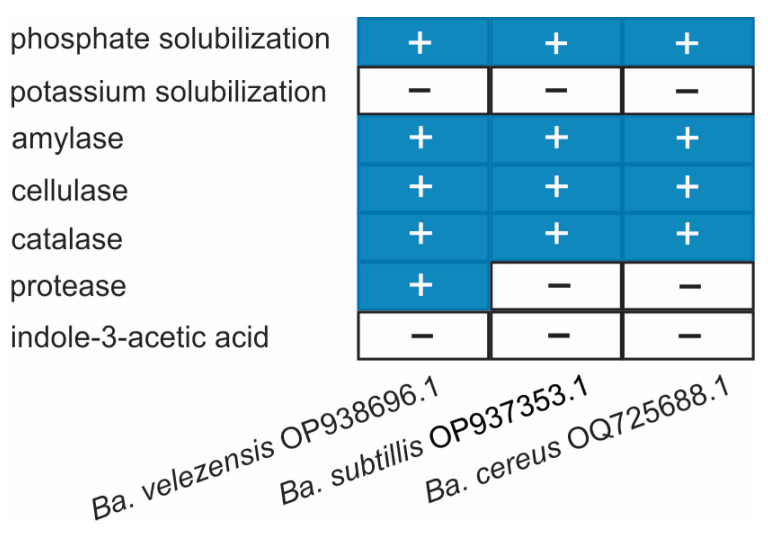
Presence (+) or absence (−) of biochemical traits in three *Bacillus* strains. Rows represent traits assessed through standard qualitative assays. Columns correspond to strains: *B. velezensis* OP938696.1, *Ba. subtilis* OP937353.1, and *Ba. cereus* OP938696.1.

Qualitative biochemical assays revealed that all strains, *Ba. velezensis* OP938696.1, *Ba. subtilis* OP937353.1, and *Ba. cereus* OP938696.1, exhibited amylolytic activity, inorganic phosphate solubilization, and catalase activity (Figure 5). These traits suggest metabolic versatility related to polysaccharide degradation, nutrient mobilization, and oxidative stress tolerance, respectively. Notably, *Ba. velezensis* also displayed cellulase and protease activity, indicating an enhanced capacity to degrade plant cell wall polymers and proteinaceous substrates, traits that may contribute to its rhizosphere competitiveness and antagonistic potential. However, none of the strains produced indole-3-acetic acid (IAA) or solubilized potassium, which may limit their role as direct plant growth promoters through phytohormone synthesis or potassium mobilization pathways.

### 2.6. Genomic Profile of Biosynthetic Gene Clusters in Bacillus spp.

**Figure 6:**
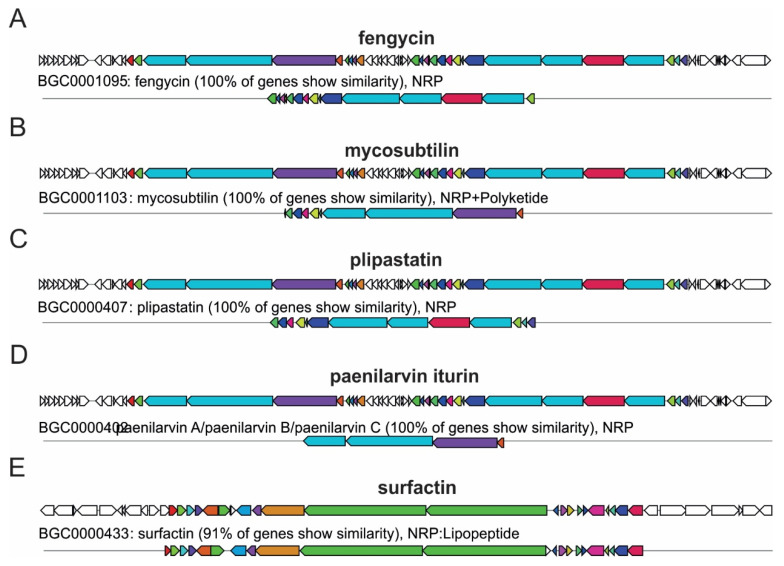
Biosynthetic gene clusters (BGCs) predicted in *Ba. velezensis* OP938696.1 using antiSMASH 7.0. Arrows represent open reading frames (ORFs), with arrow direction indicating transcription orientation. Colors denote predicted functions: non-ribosomal peptide synthetase (NRPS, red), polyketide synthase (PKS, blue), transport-related genes (green), regulatory elements (yellow), tailoring enzymes (purple), other types of enzymes (orange), and hypothetical proteins or genes with unknown functions (white) Similarity to MIBiG reference clusters: (**A**) Fengycin 100%, (**B**) Mycosubtilin 100%, (**C**) Plipastatin 100%, (**D**) Paenilarvin/Iturin 100%, and (**E**) Surfactin 91%.

Comparative genomic analysis of *Bacillus* spp. strains identified multiple biosynthetic gene clusters (BGCs) associated with the synthesis of antimicrobial secondary metabolites (Figure 6). Five BGCs exhibited high similarity to reference clusters in the MIBiG database. These included non-ribosomal peptide synthetase (NRPS)-type lipopeptide clusters responsible for the biosynthesis of fengycin, plipastatin, and paenilarvin/iturin (all 100% similarity), as well as surfactin (91%).

Additionally, a hybrid NRPS/polyketide synthase (PKS) cluster linked to mycosubtilin biosynthesis was detected with 100% similarity. These clusters displayed distinct genomic architectures comprising core NRPS and PKS biosynthetic modules, regulatory genes, accessory enzymes, transport proteins, and open reading frames (ORFs) of unknown function. The diversity of BGCs detected supports the genomic potential of *Bacillus* strains, particularly *Ba. velezensis*, to produce multiple antifungal and growth-modulating metabolites that may underlie their observed biocontrol efficacy and PGP traits.

## 3. Discussion

The use of *Bacillus* spp. represents a significant innovation in sustainable crop protection, aligning with current efforts to reduce chemical pesticide dependence through biologically based disease management. These beneficial microbes offer multifunctional advantages, combining pathogen suppression with plant growth promotion and stress tolerance enhancement. Within the context of global food security, fungal pathogens such as *Bi. oryzae* remain critical constraints to rice yield and quality, and biologically driven strategies provide a resilient, eco-compatible alternative for disease control.

Here, we provide new molecular and functional insights into the biocontrol potential of *Bacillus* species against *Bi. oryzae*, emphasizing both antagonistic efficacy and host–microbe specificity. The in vitro dual-culture assays demonstrated strong inhibition of fungal mycelial growth, with *Ba. velezensis* exhibiting over 90% inhibition, which surpasses the 67–82% inhibition previously reported for *Ba. amyloliquefaciens* against *Bipolaris* spp. [41]. This result extends the known antifungal spectrum of *Ba. velezensis*, previously established against *Fusarium oxysporum*, *Magnaporthe oryzae*, and *Xanthomonas* spp. [42,43,44], to include *Bi. oryzae*, thereby filling a notable gap in the biocontrol literature. While *Ba. velezensis*-mediated biocontrol has been reported for several pathogens, studies on rice brown spot are scarce, highlighting the novelty of the present work.

At the molecular level, genomic characterization revealed the presence of BGCs responsible for producing major lipopeptides, including fengycin, iturin, plipastatin, surfactin, and mycosubtilin, well recognized for their membrane-disruptive antifungal activity. The 100% identity with four out of five reference BGCs underscores the strain’s biosynthetic competence and corroborates previous findings linking these metabolites to inhibition of spore germination and suppression of infection structures [24,42,45]. These results highlight the genomic basis of the strain’s antagonistic capacity and provide molecular evidence supporting its application as a next-generation BCA.

Under in vivo conditions, all *Bacillus* strains effectively reduced disease severity, even under high inoculum pressure, with *Ba. velezensis* exerting the strongest effect. The protective response was more evident in the “Rubelita” cultivar, which exhibited reduced fungal colonization compared to “Predileta”. Microscopic observations revealed denser hyphal networks and abundant conidia on “Predileta”, confirming its higher susceptibility. In contrast, the restricted colonization of “Rubelita” points to partial resistance mechanisms likely involving early immune activation and localized defense responses. Previous reports have linked such genotype-dependent variations to structural barriers, secondary metabolite synthesis, and defense gene activation [3,46,47,48,49]. The consistency of these observations across independent pathosystems reinforces the hypothesis that *Bacillus*-mediated protection is modulated by host genotype.

While earlier works demonstrated *Bacillus*-mediated suppression of foliar pathogens in rice [50,51], they generally overlooked genotype-specific interactions. The present study addresses this gap, revealing significant cultivar-dependent differences in biocontrol efficacy even under standardized inoculation. Such specificity underscores the molecular dialog between the host plant and microbial biocontrol agents and points toward future genomic and transcriptomic studies to elucidate host–microbe compatibility determinants. These genotype-dependent differences underscore the need to consider host variety when developing *Bacillus*-based biocontrol strategies, ensuring optimized efficacy across cultivars.

“Rubelita” and “Predileta” are prominent Brazilian rice cultivars [52,53] with moderate resistance to brown spot. The pronounced susceptibility of “Predileta” observed here may provide a valuable reference model for pathogen–biocontrol interaction studies, particularly for assessing *Bi. oryzae* virulence and screening new antagonists. Cultivars with stable susceptibility and consistent agronomic traits are advantageous in such assays, as noted in analogous model systems [54]. Conversely, the enhanced response in “Rubelita” suggests the presence of basal defense pathways involving reactive oxygen species (ROS) signaling, MAPK activation, and SA or JA-mediated cross-talk [55,56].

An additional innovative outcome of this work is the demonstration of biomass enhancement, both shoot and root, across cultivars, even under disease pressure. This dual role of *Bacillus* strains as biocontrol and growth-promoting agents reflects their integrated impact on plant physiology. While the isolates did not exhibit typical PGP traits such as IAA synthesis or potassium solubilization, they showed strong cellulase, protease, and catalase activity. These enzymes likely contributed to nutrient cycling and oxidative stress mitigation, consistent with previous reports linking enzymatic activities to improved plant vigor under stress [57].

These findings extend prior observations in *Ba. velezensis*, where ISR and volatile organic compound (VOC)-mediated defense activation were associated with improved plant performance [43,57,58,59]. However, most studies have focused on pathogens like *M. oryzae* or *Fusarium* spp. and rarely quantified biomass responses under *Bi. oryzae* infection. The present study thus introduces new empirical evidence linking disease suppression with measurable physiological benefits, offering a bridge between molecular defense activation and whole-plant performance.

The biochemical and genomic data together suggest a multifactorial mechanism involving antifungal lipopeptide biosynthesis (fengycin, iturin, surfactin), hydrolytic enzyme secretion contributing to cell wall degradation, and indirect growth promotion via oxidative balance and nutrient mobilization. Such integrated activity is consistent with molecular characterizations of *Ba. velezensis* strains described by [24,43]. These multi-trait interactions position *Ba. velezensis* as a potent candidate for advanced biocontrol formulations combining pathogen suppression, plant resilience, and physiological enhancement.

Despite these strengths, some mechanistic gaps remain. The absence of IAA and potassium solubilization pathways limits the strains’ direct growth-promoting potential under nutrient-poor conditions [38,39,40]. However, the biomass gains observed suggest alternative PGP routes, likely mediated by enzyme-induced rhizosphere modulation [34,35]. The induction of systemic resistance through lipopeptide signaling and VOC emission, which are known to activate SA, JA, and ethylene pathways [29,31,32], may also play a key role. The consistency of biomass enhancement across both cultivars, regardless of disease resistance levels, further indicates that *Bacillus*-derived signaling may transcend genotype-specific immunity, an observation also supported by [60].

These results highlight critical future research directions. First, transcriptomic and metabolomic profiling of *Bacillus*-treated plants under *Bi. oryzae* challenge would clarify the molecular basis of ISR and growth responses, revealing cross-kingdom signaling pathways. Second, comparative genomics of *Bacillus* strains could identify unique or synergistic BGC combinations driving high antifungal efficacy. Third, field-scale validation under varying agroecological conditions is necessary to confirm the performance stability of *Ba. velezensis* and related strains. Finally, formulation development focusing on spore stability, lipopeptide preservation, and delivery efficiency could accelerate the translation of these findings into scalable bioinoculants.

Overall, this study advances the molecular and applied understanding of *Bacillus*-mediated biocontrol against *Bi. oryzae*. The integration of phenotypic, enzymatic, and genomic data provides a comprehensive framework for the development of *Ba. velezensis*-based bioformulations. By demonstrating cultivar-dependent protection, the work underscores the necessity of integrating host genetics into biocontrol design, which is an often overlooked aspect in applied microbiology. The evidence of biomass enhancement under pathogen stress expands the scope of *Bacillus* applications beyond disease control toward integrated plant resilience engineering.

In summary, the findings presented here introduce a novel, genotype-responsive, multifunctional *Bacillus* system with clear implications for both molecular plant pathology and sustainable agriculture. The high antifungal efficacy confirmed biosynthetic capacity, and physiological benefits collectively position these strains as promising candidates for next-generation biocontrol technologies. Moving forward, molecular dissection of the plant–microbe–pathogen triad, coupled with omics-driven screening and bioprocess optimization, will be critical to unlocking the full potential of *B. velezensis* as a cornerstone species for low-input, resilient rice production systems.

## 4. Materials and Methods

### 4.1. Plant Materials, Strain Origin, and Pathogen Isolation

The *Bacillus* isolates used in this study were obtained from the internal culture collection of the Molecular Biology Laboratory at the Federal University of Tocantins, Gurupi campus, Gurupi, TO, Brazil. Isolates were initially screened in a preliminary assay, and only those showing antagonistic potential relevant to the study objectives were subsequently characterized at the species level. For this, the selected isolates were chosen based on colony morphology, purified on Potato Dextrose Agar (PDA), and incubated at 28 °C for five days with daily observation. Gram staining was performed according to [61], confirming that all isolates were Gram-positive.

Rice seeds of the cultivars “Rubelita” and “Predileta” were supplied by the Department of Entomology, Federal University of Viçosa (Minas Gerais, Brazil). *Bipolaris oryzae* was isolated from naturally infected rice seeds using the Blotter test. Seeds were surface-sterilized, placed in moist chambers, and temporarily frozen to inhibit germination before re-incubation to promote fungal development. After seven days, emerging fungal colonies were transferred to PDA plates. The pathogen was identified based on its morphological characteristics, and its pathogenicity was confirmed through microculture and the fulfillment of Koch’s postulates [62].

### 4.2. In Vitro Antagonistic Bioassay

The antagonistic effect of *Bacillus* strains against *Bi. oryzae* was evaluated using the dual-culture method [63]. Each bacterial isolate was first cultured on PDA medium for 48 h at 28 °C. Subsequently, bacterial cells were aseptically transferred to 90 mm Petri dishes containing solid PDA medium. A 6 mm disk of actively growing *Bi. oryzae* culture was placed on one side of each Petri dish.

For bacterial inoculation, 0.5 µL of each standardized bacterial pre-inoculum (adjusted by optical density) was spread evenly using a platinum loop. Plates containing only the pathogen served as controls. Radial fungal growth was measured at 24, 48, 72, 96, 120, 144, and 168 h post inoculation in two perpendicular directions using a digital caliper. Four biological replicates were conducted per treatment.

The percentage of mycelial growth inhibition (I%) was calculated as follows:

I% = [(C − T)/C] × 100,

where C represents the radial growth of the fungus in the control plate (mm) and T represents the radial growth in the presence of *Bacillus* (mm) [64]. Treatments showing no inhibition were assigned a value of zero. The mean inhibition percentage was calculated per plate, which was considered the experimental unit [65].

### 4.3. Plant Biomass Assessment

Plant biomass assessment was conducted following a methodology adapted from [66]. Rice seeds were inoculated with *Bi. oryzae* conidial suspensions (1 × 10^6^ conidia per mL) and sown in seedling trays (25 cm × 30 cm) containing a sterilized mixture of sand, soil, and commercial substrate in a 1:1:1 ratio. The following treatments were applied: (i) non-inoculated control; (ii) pathogen-inoculated seeds (*Bi. oryzae* only); (iii) pathogen-inoculated seeds treated with the fungicide thiophanate; and (iv) pathogen-inoculated seeds treated with *Bacillus* spp.

Seedlings were maintained in a greenhouse at 28 °C under natural light and irrigated daily. At 55 days after emergence (DAE), corresponding to the vegetative stage (V), shoot and root tissues were collected. Fresh biomass was immediately recorded using an analytical balance. Samples were then oven-dried at 65 °C until constant weight and weighed again to determine dry biomass (g).

### 4.4. Brown Spot Severity Assessment

To assess brown spot severity, rice plants were inoculated with four concentrations of *B. oryzae* conidial suspensions: 10^6^, 10^7^, 10^8^, and 10^9^ conidia per mL. The inoculum was applied directly to the seeds prior to sowing. Plants were grown in seedling trays under greenhouse conditions (28 °C, natural light, and daily irrigation). Disease severity was evaluated at the vegetative stage using a 0–5 ordinal scale, where 0 = no visible symptoms; 1 = 1–10% leaf area affected; 2 = 11–25%; 3 = 26–50%; 4 = 51–75%; and 5 = more than 75% affected or severe necrosis. Severity was assessed on the oldest fully expanded leaf of each plant. For each treatment, ten plants per replicate were scored, and the mean severity score was calculated [67].

### 4.5. Genomic DNA Extraction and Sequencing of the B. velezensis Isolate

Genomic DNA was extracted using the DNeasy Blood and Tissue Kit (Qiagen, Hilden, Germany), following the manufacturer’s instructions. DNA quality and concentration were verified by spectrophotometry and agarose gel electrophoresis to ensure sample integrity. The extracted DNA was randomly fragmented into short segments by ultrasonication (Covaris). The resulting fragments were end-repaired, A-tailed, and ligated with Illumina sequencing adapters. Adapter-ligated fragments were size-selected, PCR-amplified, and purified prior to sequencing.

Raw reads underwent quality control using FastQC v0.11.9 (https://www.bioinformatics.babraham.ac.uk/projects/fastqc/ (accessed on 2 February 2024)) and Trim Galore (https://github.com/FelixKrueger/TrimGalore (accessed on 2 February 2024)) for adapter and low-quality base removal. Genome assembly was performed with SPAdes v3.15.5 using the -isolate parameter [68]. Contigs with k-mer coverage lower than 20 were filtered out using SeqKit v.2.12 [69] and csvtk v0.36 (https://github.com/shenwei356/csvtk (accessed on 2 February 2024)).

Species-level identification and phylogenetic analysis were conducted via digital DNA-DNA hybridization (dDDH) on the Type Strain Genome Server (TYGS) [70] Genome completeness was evaluated using BUSCO v5.4.3, employing the orthologous gene set for the order *Bacillales*. Plasmid content was predicted using Mob-suite v2.0 [71].

Genome annotation was performed with Prokka v1.14.6 [72]. The FASTA file containing predicted protein sequences was analyzed using the BlastKOALA server (https://www.kegg.jp/blastkoala/ (accessed on 2 February 2024)) to assign KEGG Orthology (KO) identifiers [73]. KO numbers were subsequently used to reconstruct metabolic pathways via KEGG Mapper (https://www.genome.jp/kegg/mapper/ (accessed on 2 February 2024)) [73]. Biosynthetic gene clusters (BGCs) associated with antifungal compound synthesis were predicted and annotated using AntiSMASH for bacteria [74].

The genome of *B. velezensis* strain described in publicly available in Genbank under accession number JBSZEF000000000.

### 4.6. Assays of Enzymatic and Biochemical Activity

The biochemical characteristics of *Bacillus* spp. isolates were determined using standard qualitative assays to assess their potential PGP activities.

- Amylase activity was evaluated on starch agar medium (10 g/L peptone, 5 g/L yeast extract, 5 g/L NaCl, 2 g/L starch, and 15 g/L agar; pH 6.9) following [75]. After incubation, plates were flooded with Lugol’s iodine solution for 30 min, and the presence of a clear halo around colonies indicated positive activity.
- Protease activity was assessed on skim milk agar (3% *v*/*v*), incubated at 28 °C for 48 h. Transparent zones around colonies were considered indicative of proteolytic enzyme production [76].
- Phosphate solubilization was determined according to [77]. Isolates were cultured in phosphate-supplemented liquid medium at 28 ± 2 °C with shaking (150 rpm) for 72 h. Solubilized phosphorus was quantified by using the Murphy–Riley method [78], where a color shift from purple to yellow indicated a positive result.
- IAA production was tested in Luria–Bertani (LB) broth supplemented with 100 mg/L tryptophan. Cultures were incubated at 28 °C for five days with shaking at 200 rpm. For detection, 1 mL of culture supernatant was mixed with 2 mL of Salkowski reagent (2% FeCl_3_·6H2O in 37% HClO_4_) and incubated in the dark for 12 h. A pink to reddish coloration indicated IAA production [79].
- Catalase activity was determined by adding 1 mL of 3% H_2_O_2_ directly to agar-grown cultures previously incubated at 28 °C for 48 h in LB medium (pH 7.0). The immediate formation of oxygen bubbles confirmed a positive reaction [80].
- Cellulase activity was evaluated on minimal medium (MM) supplemented with 1% carboxymethylcellulose (1 g glucose, 2.5 g yeast extract, 15 g agar per liter). After 48 h of incubation at 28 °C, plates were stained with Congo red dye. The appearance of clear yellow halos around colonies indicated cellulase production [81].

### 4.7. Statistical Analysis

Data on mycelial growth inhibition were analyzed via regression using SigmaPlot v12.0. Model fit was evaluated based on the coefficient of determination (R^2^) and residual distribution. Shoot and root biomass data were subjected to ANOVA followed by Duncan’s multiple range test for mean separation (SAS v9.0). Statistical significance was considered at *p* < 0.05 for all analyses.

## 5. Conclusions

The *Bacillus* spp. strains evaluated in this study, particularly *Bacillus velezensis*, exhibited strong and consistent biocontrol activity against *Bi. oryzae*, the causal agent of rice brown spot. Under both in vitro and in vivo conditions, *Ba. velezensis* achieved high levels of fungal inhibition and markedly reduced disease severity, while also enhancing shoot and root biomass even under pathogen pressure.

The contrasting responses observed between the rice cultivars “Predileta” and “Rubelita” highlight the critical role of host genotype in shaping plant–microbe–pathogen interactions. Rubelita’s reduced colonization and lower disease severity indicate a more tolerant phenotype, suggesting that host genetic background influences both infection dynamics and biocontrol efficacy.

Although the strains did not express classical plant growth-promoting traits such as IAA production or potassium solubilization, their synthesis of antifungal metabolites and secretion of hydrolytic enzymes support a multifactorial mode of action. This mechanism likely integrates direct pathogen suppression with indirect physiological stimulation through enhanced stress tolerance, nutrient mobilization, and ISR.

Altogether, these results demonstrate that native *Bacillus* strains, especially *Ba. velezensis*, are valuable resources for sustainable rice protection. Their dual capacity to control disease and promote plant vigor positions them as promising candidates for eco-friendly, low-input management programs. As the molecular basis for the genotype-specific responses observed here remains unclear, future transcriptomic and proteomic approaches will be important to elucidate the defense pathways activated in each genotype and the regulatory networks underlying antifungal metabolite production. Field-scale validation will also be essential to assess consistency across diverse environments.

In general, this study provides a foundation for the development of genotype-responsive, biologically based crop protection systems aligned with global efforts to reduce chemical pesticide dependency.

## Data Availability

The original contributions presented in this study are included in the article. Further inquiries can be directed at the corresponding authors.

## Supplementary Materials

The following supporting information can be downloaded at: https://www.mdpi.com/article/10.3390/ijms27031455/s1.
