# Supplementary material for: Bacillus velezensis Enhances Rice Resistance to Brown Spot by Integrating Antifungal and Growth Promotion Functions
Source: Int J Mol Sci. 2026 Feb 1;27(3):1455. doi: 10.3390/ijms27031455 (PMC12898680; doi:10.3390/ijms27031455)
Supplement: Supplementary file 1 [file ijms-27-01455-s001.zip › ijms-4017375-supplementary.pdf]

Supplementary Materials

Bacillus velezensis Enhances Rice Resistance to Brown Spot by Integrating Antifungal and Growth Promotion Functions

Elizabeth B. E. Pires[1#], Maira S. Tique Obando[1#], Luís J. Maia[1,2], Bergmann M. Ribeiro[2], Odaiza F. Souza[1], Marcelo L. Dias[3], Luís O. Viteri Jumbo[1,4], Rodrigo R. Fidelis[4], Gil. R. Santos[1,4], Raimundo N. C. Rocha[5], Guy Smagghe[1,6,7*], Tito Bacca[8], Eugenio E. Oliveira[1,9*], Rudolf Haumann[10], Raimundo W. S. Aguiar[1,3,4]

1 Programa de Pós-Graduação em Biotecnologia, Universidade Federal do Tocantins (UFT), Gurupi 77402-970, TO, Brazil
2 Departamento de Biologia Celular, Universitdade de Brasília, Brasília 70297-400, DF, Brazil
3 Programa de Pós-graduação em Biodiversidade e Biotecnologia – Rede Bionorte, Universidade Federal do Tocantins (UFT), Gurupi, TO, 77402-970, Brazil
4 Programa de Pós-graduação em Produção Vegetal, Universidade Federal do Tocantins, Gurupi 77410-530, TO, Brazil
5 Embrapa Arroz e Feijão, Santo Antônio de Goiás 75375-000, GO; Brazil
6 Institute of Entomology, Guizhou University, Guiyang 550025, China
7 Department of Biology, Vrije Universiteit Brussel (VUB), 1050 Brussels, Belgium;
8 Facultad de Ingeniería Agronómica, Universidad del Tolima, Ibagué 730001, Tolima, Colombia;
9 Departamento de Entomologia, Universidade Federal de Viçosa, Viçosa 36570-900, MG, Brazil;
10 Department of Bioprocess Engineering, Institute of Food Science and Biotechnology, University of Hohenheim, 70599 Stuttgart, Germany.
# These authors contributed equally for the manuscript
* Authors to whom correspondence should be addressed.

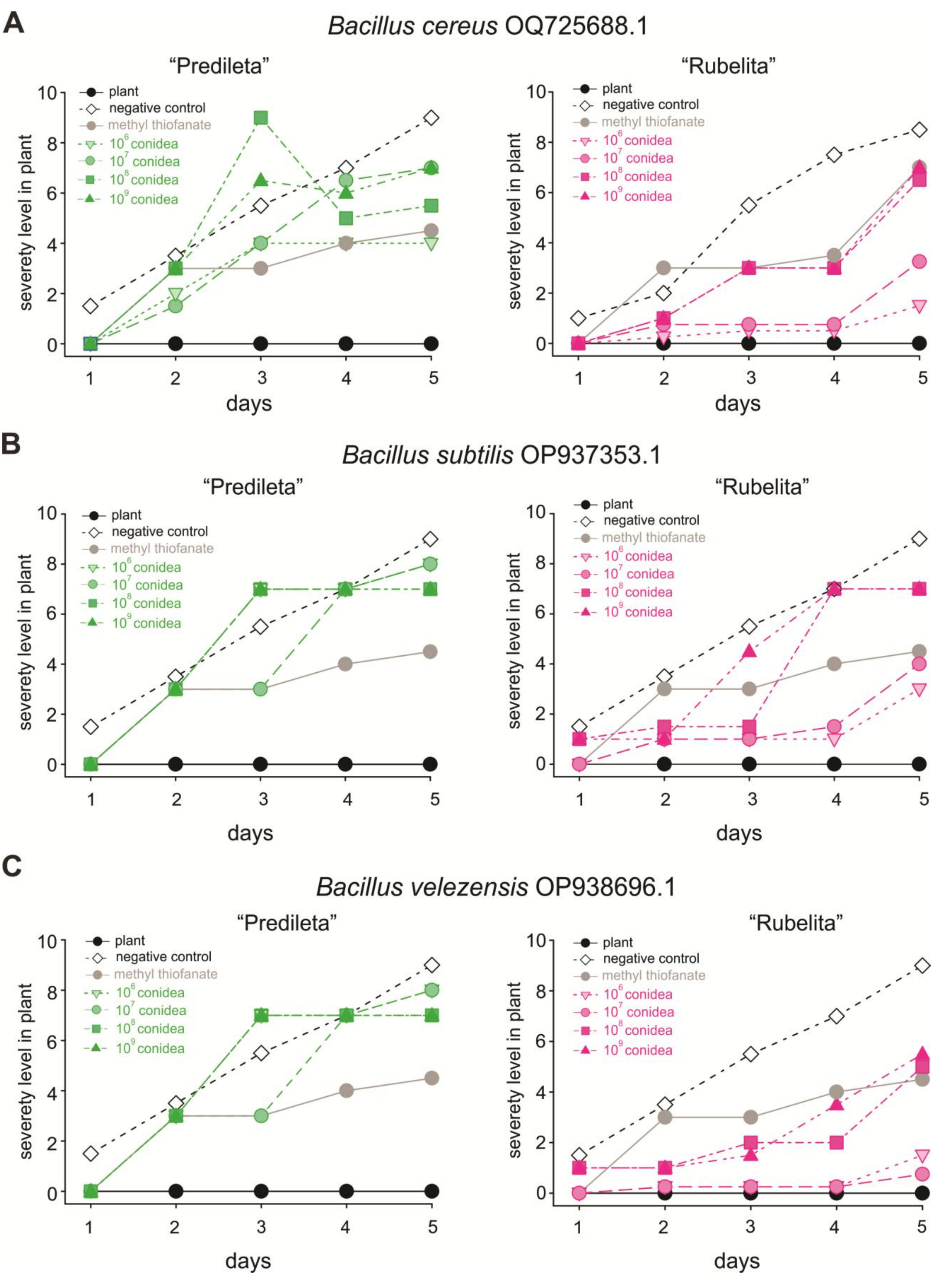

Supplementary Figure S1. Severity of brown spot disease in rice plants of the "Predileta" and "Rubelita" cultivars treated with *Bacillus cereus* OQ725688.1 (***A***), *Bacillus subtilis* OP937353.1 (***B***), and *Bacillus velezensis* OP938696.1 (***C***). Symbols showed the means of four replicates.

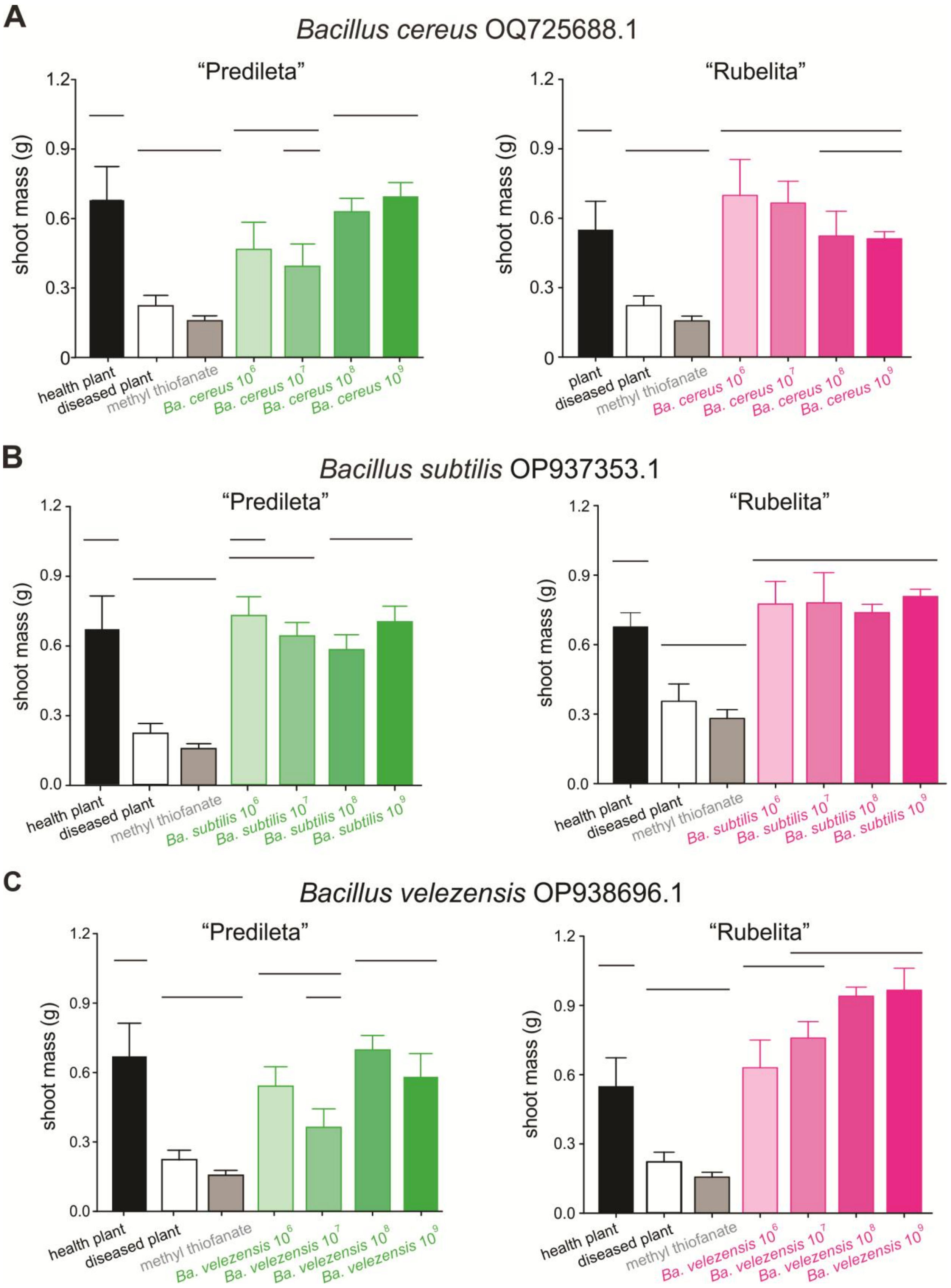

Supplementary Figure S2. Shoot biomass (g) of "Predileta" (A-C, left) and "Rubelita" (D-F, right) rice seedlings following treatment with *Bacillus* spp. Bars represent mean ± SE of four replicates. Horizontal lines above bars indicate no significant differences (Duncan test, $P < 0.05$).

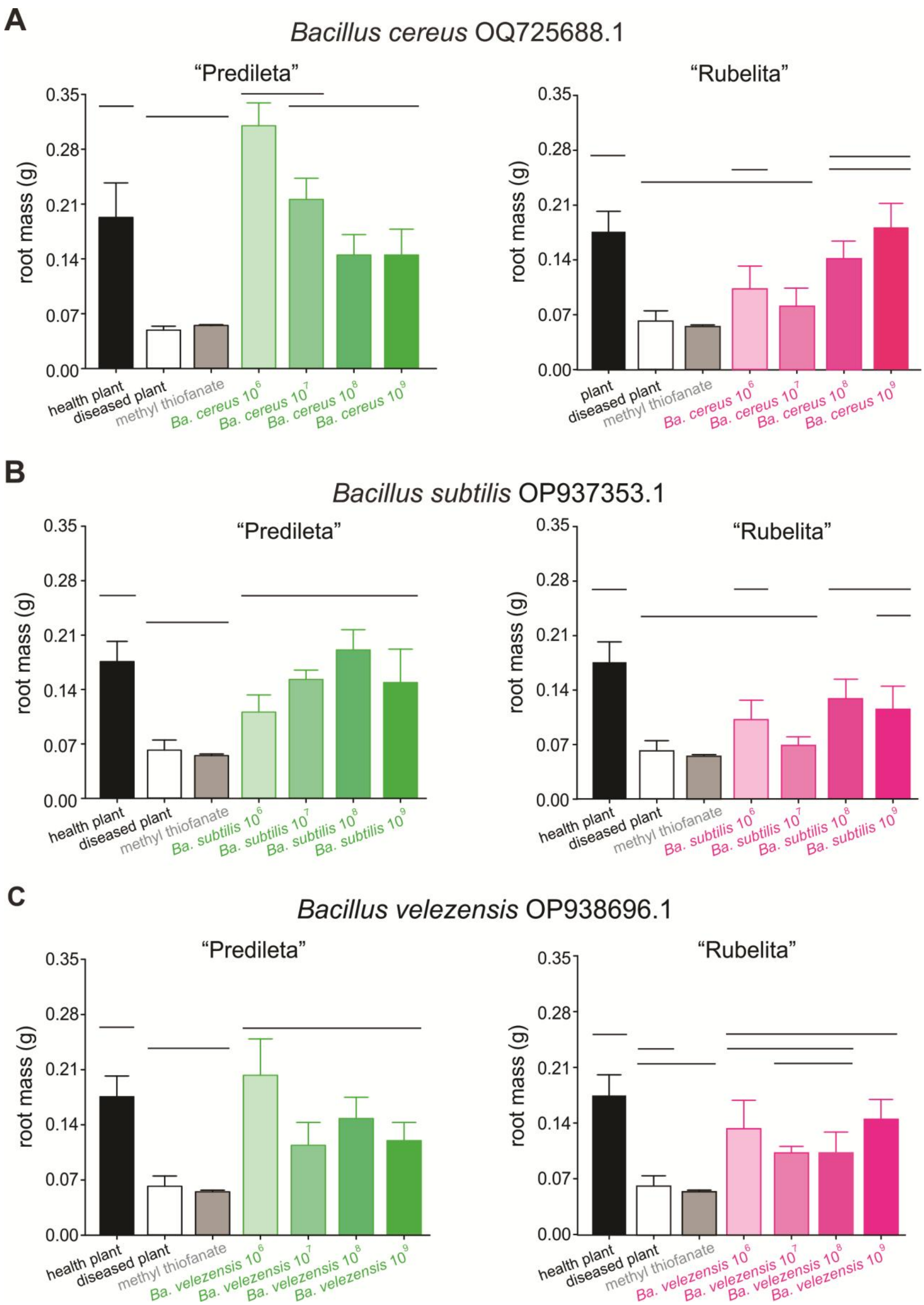

Supplementary Figure S3. Root biomass (g) of "Predileta" (A-C, left) and "Rubelita" (D-F, right) rice seedlings after *Bacillus* spp. treatment. Bars represent mean ± SE of four replicates; horizontal lines indicate no significant differences (Duncan test, $P < 0.05$).
